# Association of Coded Housing Instability and Hospitalization in the US

**DOI:** 10.1001/jamanetworkopen.2022.41951

**Published:** 2022-11-14

**Authors:** Kimberly A. Rollings, Nicholas Kunnath, Caitlin R. Ryus, Alexander T. Janke, Andrew M. Ibrahim

**Affiliations:** 1 Health and Design Research Fellowship Program, Institute for Healthcare Policy and Innovation, University of Michigan, Ann Arbor; 2 Department of Surgery, University of Michigan, Ann Arbor; 3 Center for Healthcare Outcomes and Policy, University of Michigan, Ann Arbor; 4 Department of Emergency Medicine, Yale School of Medicine, New Haven, Connecticut; 5 Veterans Affairs Health Services Research and Development Center for the Study of Healthcare Innovation, Implementation, and Policy/Institute for Healthcare Policy and Innovation, University of Michigan, Ann Arbor; 6 Taubman College of Architecture and Urban Planning, University of Michigan, Ann Arbor

## Abstract

**Importance:**

Housing instability and other social determinants of health are increasingly being documented by clinicians. The most common reasons for hospitalization among patients with coded housing instability, however, are not well understood.

**Objective:**

To compare the most common reasons for hospitalization among patients with and without coded housing instability.

**Design, Setting, and Participants:**

This cross-sectional, retrospective study identified hospitalizations of patients between age 18 and 99 years using the 2017 to 2019 National Inpatient Sample. Data were analyzed from May to September 2022.

**Exposures:**

Housing instability was operationalized using 5 *International Classification of Diseases, 10th Revision, Social Determinants of Health Z-Codes* addressing problems related to housing: homelessness; inadequate housing; discord with neighbors, lodgers, and landlords; residential institution problems; and other related problems.

**Main Outcomes and Measures:**

The primary outcome of interest was reason for inpatient admission. Bivariate comparisons of patient characteristics, primary diagnoses, length of stay, and hospitalization costs among patients with and without coded housing instability were performed.

**Results:**

Among the 87 348 604 hospitalizations analyzed, the mean (SD) age was 58 (20) years and patients were more likely to be women (50 174 117 [57.4%]) and White (58 763 014 [67.3%]). Housing instability was coded for 945 090 hospitalizations. Hospitalized patients with housing instability, compared with those without instability, were more likely to be men (668 255 patients with coded instability [70.7%] vs 36 506 229 patients without [42.3%]; *P* < .001), younger (mean [SD] age 45.5 [14.0] vs 58.4 [20.2] years), Black (235 355 patients [24.9%] vs 12 929 158 patients [15.0%]), Medicaid beneficiaries (521 555 patients [55.2%] vs 15 541 175 patients [18.0%]), uninsured (117 375 patients [12.4%] vs 3 476 841 patients [4.0%]), and discharged against medical advice (28 890 patients [8.4%] vs 451 855 patients [1.6%]). The most common reason for hospitalization among patients with coded housing instability was mental, behavioral, and neurodevelopmental disorders (475 575 patients [50.3%]), which cost a total of $3.5 billion. Other common reasons included injury (69 270 patients [7.3%]) and circulatory system diseases (64 700 patients [6.8%]). Coded housing instability was also significantly associated with longer mean (SD) hospital stays (6.7 [.06] vs 4.8 [.01] days) and a cost of $9.3 billion. Hospitalized patients with housing instability had 18.6 times greater odds of having a primary diagnosis of mental, behavioral, and neurodevelopmental disorders (475 575 patients [50.3%] vs 4 470 675 patients [5.2%]; odds ratio, 18.56; 95% CI, 17.86 to 19.29).

**Conclusions and Relevance:**

In this cross-sectional study, hospitalizations among patients with coded housing instability had higher admission rates for mental, behavioral, and neurodevelopmental disorders, longer stays, and increased costs. Findings suggest that efforts to improve housing instability, mental and behavioral health, and inpatient hospital utilization across multiple sectors may find areas for synergistic collaboration.

## Key Points

**Question:**

What are the most common reasons for hospitalization among patients with coded housing instability?

**Findings:**

This cross-sectional study of 87 348 604 hospitalizations found that the most common reasons for hospitalization among patients with coded housing instability were mental, behavioral, and neurodevelopmental disorders (50.3%), injury (7.3%), and circulatory system diseases (6.8%). Coded housing instability was also significantly associated with longer hospital stays (6.7 vs 4.8 days) and a cost of $9.3 billion.

**Meaning:**

These findings suggest that coded housing instability was associated with higher rates of inpatient admissions for mental, behavioral, and neurodevelopmental disorders, longer hospital stays, and substantial health care costs.

## Teaser

This cross-sectional study examines the association between coded housing instability and hospitalization in the US.

## Introduction

Housing instability is a social determinant of health (SDOH) increasingly being addressed by hospitals, health systems, and payers.^1,2,3,4,5^ Variable definitions of housing instability refer to several housing-related problems, such as difficulty paying rent, spending a high proportion of income on housing, moving frequently, overcrowding, and staying with relatives.^6,7^ Housing instability exists on a continuum and includes the experience of homelessness.^6^ With increasing US rates of housing instability and attention to SDOH,^8^ federal and state payers (eg, Medicare and Medicaid), managed care organizations, and hospitals are investing in local efforts to create and preserve affordable housing.^1,8,9,10^

Despite broad interest in housing instability and use of inpatient hospital resources, the relationship remains understudied. Prior work has raised concern that patients with housing instability present with more serious infections, sequalae of preventable exposures, and health crises due to untreated chronic conditions, such as diabetes, high blood pressure, and heart disease.^4,11^ Housing instability has also been associated with poor primary health care utilization, as patients often delay care due to cost.^12,13^ Previous studies^14,15,16,17,18,19,20,21,22^ indicated that, as a consequence, patients experiencing housing instability have higher rates of costly hospitalization, readmission, and emergency department service use compared with patients without housing instability. Many of these studies, however, only included specific health care services, used nonrepresentative data, examined a short period of time, or focused exclusively on homelessness and not multiple levels of housing instability.^23^ As such, understanding of how patients with housing instability use inpatient hospital resources is incomplete.

In this context, this study aimed to identify the most common diagnoses, length of stay, and associated costs among hospitalized patients experiencing housing instability. To achieve this aim, the largest publicly available, all-payer inpatient health care database and several housing instability indicators coded by clinicians via *International Statistical Classification of Diseases and Related Health Problems, Tenth Revision (ICD-10), Social Determinants of Health Z-Codes* were used. This study is timely as health care systems with large inpatient practices explore ways to invest in communities and address SDOH, including housing instability.

## Methods

### Data Source and Participants

This cross-sectional study was exempt from approval and the need for informed consent by the University of Michigan institutional review board because a publicly available data set containing deidentified patient information was used. The Strengthening the Reporting of Observational Studies in Epidemiology (STROBE) reporting guidelines were followed.

This cross-sectional, retrospective study used the National Inpatient Sample (NIS) between January 2017 and December 2019. The NIS is part of the Healthcare Cost and Utilization Project sponsored by the Agency for Healthcare Research and Quality.^24^ The database contains deidentified information about each hospitalization, including patient and hospital demographics, admission status, discharge diagnoses, comorbidities, procedures, outcomes, costs, and payer information. Unweighted, the NIS data contain a 20% sample of all inpatient discharges from US community hospitals (>7 million hospitalizations annually; enabling weighted estimates of >35 million hospitalizations nationally), making it the largest publicly available all-payer inpatient care database. Approximately 85% of US hospitals are community hospitals, including all nonfederal, short-term, general, and other specialty hospitals. Rehabilitation, psychiatric, and long-term acute care hospitals, as well as hospital units of institutions, are excluded from the NIS. Detailed information about the NIS sampling methods and design are available elsewhere.^24^ Admissions among patients aged 18 to 99 years with complete study data were included.

### Exposure

Screening patients for housing instability and other SDOH is not consistent or standardized.^25,26^ In this study, housing instability was operationalized using 5 *ICD-10* Z-Codes addressing aspects of the housing instability continuum, including but not exclusive to homelessness: homelessness (Z59.0); inadequate housing (Z59.1); discord with neighbors, lodgers, and landlords (Z59.2); problems related to living in a residential institution (Z59.3); and other problems relating to housing and economic circumstances (Z59.8: housing instability, housed: with risk of homelessness; homelessness in past 12 months, unspecified; other problems related to housing and economic circumstances; and problem related to housing and economic circumstances, unspecified). Consistent with other work,^27,28^ this study reviewed and identified coded housing instability for hospitalizations with at least 1 of the 5 housing-related Z59-codes in the first 15 *ICD-10* codes in the NIS data set. Patient-level characteristics included age (continuous variable), sex, race and ethnicity (Asian, Black, Hispanic, American Indian, White, and other, including multiple races and state-reported race categories excluded from other NIS categories), insurance type (Medicare, Medicaid, private, uninsured, other), elective or emergency admittance, major operating room procedure, and discharge type (routine transfer to short term hospital, other transfer, home health care, against medical advice). Race and ethnicity were reported to examine whether rates of coded housing instability differed by these factors.

### Main Outcomes and Measures

The main outcome of interest was the primary reason for hospitalization. Diagnoses and procedures are recorded in the NIS database using *International Statistical Classification of Diseases, Tenth Revision, Clinical Modification (ICD-10-CM)* codes for hospitalizations after October 1, 2015. Diagnosis groups were created based on the first 3 digits of the *ICD-10-CM* primary diagnosis codes (eTable 1 in the Supplement).

Total inpatient days were calculated by summing across admissions and diagnosis groups. Mean length of stay was calculated by dividing total inpatient days by the number of admissions. Same-day stays are coded as 0 in the NIS data.

Hospitalization costs were calculated using NIS charge data and Healthcare Cost and Utilization Project cost-to-charge ratio files. After merging the 2 data sets by year and NIS charge data by NIS hospital ID, charges were multiplied by the cost-to-charge ratio. Costs in 2017 and 2018 were adjusted for 2019 inflation.

### Statistical Analysis

The goal of this analysis was to examine the association between coded housing instability and reason for inpatient hospitalization, length of stay, and costs of admission. Standard descriptive statistics were first calculated to characterize hospitalizations overall and by coded housing instability status. Procedures that incorporated survey sampling weights to account for the complex NIS sampling design and to provide representative estimates of the national US population were applied. Diagnoses, total inpatient days, mean length of stay, and annual hospitalization costs were also similarly calculated. The most common reasons for hospitalization among patients with and without coded housing instability were identified and compared via estimation of unadjusted odds ratios (ORs). Due to the large sample size, standardized differences were used to examine effect sizes (Cohen *d*) of the differences between groups with and without coded housing instability, independent of sample size. Standardized differences were calculated for comparisons of coded housing instability status by age, sex, race and ethnicity, insurance type, elective or emergency admittance, discharge type, primary discharge diagnosis, total inpatient days, mean length of stay, and annual costs. Resulting effect sizes were assessed according to 3 thresholds: small (0.20-0.49), medium (0.50-0.79), and large (≥0.80) effect sizes.^29^ Additional sensitivity analyses were performed and examined all comparisons stratified by the 5 housing instability Z59 codes. All analyses were performed using STATA statistical software version 17 (STATACorp). Data were analyzed from May to September 2022.

## Results

Analysis of the 87 348 604 hospitalizations included in the study indicated that patients were mean (SD) age of 58 (20) years, more likely to be women (50 174 117 [57.4%]), and mostly of White race (58 763 014 [67.3%]). Details on selection and exclusion criteria are presented in eTable 2 in the Supplement. Table 1 summarizes these and additional characteristics of hospitalizations between 2017 to 2019. Overall, patients were also primarily Medicare beneficiaries (48.3% [42 174 988]), admitted by emergency (61.9% [54 035 986]), and routinely discharged (62.8% [18 321 765]). This study identified 945 090 admissions with at least 1 of 5 housing instability codes. Admissions with coded housing instability were more likely to be men compared with the 86 403 514 admissions without coded housing instability (70.7% [668 255] vs 42.3% [36 506 229]), younger (mean [SD] age 45.5 [14.0] years vs 58.4 [20.2] years), Black (24.9% [235 355] vs 15.0% [12 929 158]), Medicare (21.0% [198 070] vs 48.6% [41 976 918]) and Medicaid beneficiaries (55.2% [521 555] vs 18.0% [15 541 175]), uninsured (12.4% [117 375] vs 4.0% [3 476 841]), admitted by emergency (75.2% [710 635] vs 61.7% [53 325 351]), and discharged against medical advice (8.4% [28 890] vs 1.6% [451 855]). Among admissions for patients with coded housing instability, 96.8% had the Z59.0 homelessness code.

**Table 1.** Characteristics of Hospitalized Patients With and Without Coded Housing Instability^a^

Characteristic	Patient admissions, No. (%)			Standardized difference (Cohen *d*)^c^
	Total admissions (N = 87 348 601)	With coded housing instability admissions (n = 945 090)^b^	Without coded housing instability admissions (n = 86 403 511)	
Age, mean (SD), y	58.3 (20.2)	45.5 (14.0)	58.4 (20.2)	0.642
Sex				
Men	37 174 484 (42.6)	668 255 (70.7)	36 506 229 (42.3)	0.577
Women	50 174 117 (57.4)	276 835 (29.3)	49 897 282 (57.7)	0.577
Race				
Asian or Pacific Islander	2 441 628 (2.8)	11 920 (1.3)	2 429 708 (2.8)	0.094
Black	13 164 513 (15.1)	235 355 (24.9)	12 929 158 (15.0)	0.278
Hispanic	9 806 187 (11.2)	108 320 (11.5)	9 697 867 (11.2)	0.008
American Indian	566 015 (0.6)	12 895 (1.4)	553 120 (0.6)	0.090
White	58 763 014 (67.3)	544 525 (57.6)	58 218 489 (67.4)	0.208
Other^d^	2 607 244 (3.0)	32 075 (3.4)	2 575 169 (3.0)	0.024
Insurance type				
Private	23 071 974 (26.4)	72 125 (7.6)	22 999 849 (26.6)	0.431
Medicare	42 174 988 (48.3)	198 070 (21.0)	41 976 918 (48.6)	0.554
Medicaid	16 062 729 (18.4)	521 555 (55.2)	15 541 175 (18.0)	0.965
Other^d^	2 444 694 (2.8)	35 965 (3.8)	2 408 729 (2.8)	0.062
Uninsured	3 594 216 (4.1)	117 375 (12.4)	3 476 841 (4.0)	0.423
Elective procedure	19 855 840 (22.7)	69 160 (7.3)	19 786 680 (22.9)	0.372
Emergency admittance	54 035 986 (61.9)	710 635 (75.2)	53 325 351 (61.7)	0.278
Major operating room procedure	9 206 992 (31.6)	27 270 (7.9)	9 179 722 (31.8)	0.516
Discharge description				
Routine	18 321 765 (62.8)	261 730 (75.7)	18 060 035 (62.7)	0.269
Transfer to short-term hospital	586 409 (2.0)	5980 (1.7)	580 429 (2.0)	0.020
Other transfer	4 778 945 (16.4)	42 430 (12.3)	4 736 515 (16.4)	0.113
Home health care	4 350 181 (14.9)	5985 (1.7)	4 344 196 (15.1)	0.375
Against medical advice	480 745 (1.6)	28 890 (8.4)	451 855 (1.6)	0.534
Housing instability Z59-codes^e^				
Z59.0 homelessness	NA	915 305 (96.8)	NA	NA
Z59.1 inadequate housing	NA	5030 (0.5)	NA	NA
Z59.2 discord with neighbors, lodgers, landlords	NA	1220 (0.1)	NA	NA
Z59.3 problems related to living in a residential institution	NA	3335 (0.4)	NA	NA
Z59.8 other problems	NA	24 445 (2.6)	NA	NA

Abbreviations: NA, not applicable; NIS, National Inpatient Sample.

^a^ Data source: National Inpatient Sample, 2017 to 2019.

^b^ “With coded housing instability” data reflect hospitalizations with at least 1 of 5 housing instability Z59-codes.

^c^ “Standardized difference” displays the absolute value of the difference in proportions divided by the standard error and is an indicator of effect size (Cohen *d*) (0.20-0.49 indicates small; 0.50 to 0.79, medium; and ≥0.80, large effect sizes).

^d^ Other race includes multiple races and state-reported race categories excluded from other NIS categories.

^e^ “Housing instability Z59-codes” display the number of hospitalizations with each of the 5 listed Z59-codes.

There were significant differences in the most common reasons for hospitalization among patients with and without coded housing instability (Table 2). Admissions with coded housing instability had 18.6 times greater odds of having a mental, behavioral, and neurodevelopmental disorder diagnosis (50.3% [475 575] vs 5.2% [4 470 675]; OR, 18.56; 95% CI = 17.86-19.29) (Table 2 and eFigure in the Supplement). The second and third most common reasons for hospitalization among admissions with coded housing instability were injury (7.3% [69 270] vs 8.7% [7 559 439], OR = 0.82; 95% CI, 0.80-0.86), and diseases of the circulatory system (6.8% [64 700] vs 16.7% [14 452 895 ], OR, 0.37; 95% CI, 0.35-0.38). For comparison, the 3 most common reasons for admissions among patients without coded housing instability were diseases of the circulatory system (16.6% [14 452 895]), pregnancy, childbirth, and puerperium (12.8% [11 157 270]), and diseases of the digestive system (9.9% [8 581 113]).

**Table 2.** Hospital Admissions by Diagnosis Among Patients With and Without Coded Housing Instability^a^

Discharge diagnosis	Patient admissions, No. (%)			Odds ratio (95% CI)	Standardized difference (Cohen *d*)^b^
	Total admissions	With coded housing instability admissions	Without coded housing instability admissions		
Mental, behavioral, and neurodevelopmental disorders	4 946 250 (5.7)	475 575 (50.3)	4 470 675 (5.2)	18.56 (17.86-19.29)	1.994
Injury, poisoning, and certain other consequences of external causes	7 628 709 (8.7)	69 270 (7.3)	7 559 439 (8.7)	0.82 (0.80-0.86)	0.050
Diseases of the circulatory system	14 517 595 (16.6)	64 700 (6.8)	14 452 895 (16.7)	0.37 (0.35-0.38)	0.266
Certain infections and parasitic diseases	7 184 214 (8.2)	56 060 (5.9)	7 128 154 (8.2)	0.70 (0.67-0.73)	0.084
Diseases of the respiratory system	6 970 298 (8.0)	51 365 (5.4)	6 918 933 (8.0)	0.66 (0.64-0.68)	0.095
Diseases of the skin and subcutaneous tissue	1 652 514 (1.9)	46 665 (4.9)	1 605 849 (1.9)	2.74 (2.64-2.85)	0.226
Endocrine, nutritional, and metabolic diseases	3 800 334 (4.4)	38 710 (4.1)	3 761 624 (4.4)	0.94 (0.91-0.97)	0.013
Diseases of the digestive system	8 618 458 (9.9)	37 345 (4.0)	8 581 113 (9.9)	0.37 (0.36-0.39)	0.201
Diseases of the musculoskeletal system and connective tissue	6 259 779 (7.2)	19 310 (2.0)	6 240 469 (7.2)	0.27 (0.26-0.28)	0.201
Diseases of the genitourinary system	4 103 464 (4.7)	18 030 (1.9)	4 085 434 (4.7)	0.39 (0.38-0.41)	0.133
Symptoms, signs, and abnormal clinical laboratory findings, not elsewhere classified	2 419 310 (2.8)	17 910 (1.9)	2 401 400 (2.8)	0.68 (0.65-0.70)	0.054
Diseases of the nervous system	2 243 755 (2.6)	17 270 (1.8)	2 226 485 (2.6)	0.70 (0.67-0.73)	0.047
Pregnancy, childbirth, and puerperium	11 174 720 (12.8)	17 450 (1.8)	11 157 270 (12.9)	0.13 (0.12-0.13)	0.332
Neoplasms	3 566 719 (4.1)	7100 (0.8)	3 559 619 (4.1)	0.18 (0.17-0.19)	0.170
Diseases of the blood and blood-forming organs and certain disorders involving the immune mechanism	1 079 290 (1.2)	4420 (0.5)	1 074 870 (1.2)	0.37 (0.35-0.40)	0.070
Factors influencing health status and contact with health services	911 070 (1.0)	2415 (0.3)	908 655 (1.1)	0.24 (0.21-0.27)	0.078
Diseases of the eye and adnexa	66 715 (0.1)	895 (0.1)	65 820 (0.1)	1.24 (1.07-1.45)	0.007
Certain conditions originating in the perinatal period^c^	905 (<0.01)	0	905 (<0.01)	NA	NA
Congenital malformations, deformations, and chromosomal abnormalities	109 755 (0.1)	225 (0.0)	109 530 (0.1)	0.19 (0.14-0.25)	0.029
Diseases of the ear and mastoid process	94 350 (0.1)	370 (<0.01)	93 980 (0.1)	0.36 (0.29-0.45)	0.021
External causes of morbidity	400 (<0.01)	5 (<0.01)	395 (<0.01)	1.16 (0.18-7.54)	0.000
Total No. of hospital admissions	87 348 604 (100)	945 090 (1.1)	86 403 514 (98.9)	NA	NA

Abbreviation: NA, not applicable.

^a^ Data source: National Inpatient Sample, 2017 to 2019.

^b^ Standardized difference displays the absolute value of the difference in proportions divided by the standard error and is an indicator of effect size (Cohen *d*) (>0.20 indicates small; >0.50, medium; and ≥.80, large effect sizes).

^c^ Certain conditions originating in the perinatal period had no coded housing instability so no odds ratio or standardized difference is reported.

Table 3 presents total inpatient days for hospitalizations with and without coded housing instability by diagnosis and overall mean length of stay. Hospitalizations with coded housing instability accounted for 1.5% (6 197 196) of the 416 199 050 total inpatient days between 2017 to 2019. When compared with hospitalizations without coded housing instability, admissions with coded housing instability had a greater mean (SD) length of stay (6.7 [.06] days vs 4.8 [.01] days). Of the 6 197 196 inpatient days among those with coded housing instability, 59.1% (3 661 816) were due to mental, behavioral, and neurodevelopmental disorders compared with 7.6% (31 111 715) among patients without coded housing instability. Diseases of the circulatory system accounted for the highest percentage of inpatient days among admissions without coded housing instability (17.4% [71 429 857]).

**Table 3.** Total Inpatient Days and Mean Length of Stay by Diagnosis Among Patients With and Without Coded Housing Instability^a^

Discharge diagnosis	Days, No. (%)			Standardized difference (Cohen *d*)^b^
	Total	With coded housing instability	Without coded housing instability	
Mental, behavioral, and neurodevelopmental disorders	34 773 530 (8.4)	3 661 816 (59.1)	31 111 715 (7.6)	1.920
Injury, poisoning, and certain other consequences of external causes	43 386 087 (10.4)	408 225 (6.6)	42 977 862 (10.5)	0.127
Certain infections and parasitic diseases	49 113 167 (11.8)	384 130 (6.2)	48 729 037 (11.9)	0.176
Diseases of the circulatory system	71 780 192 (17.2)	350 335 (5.7)	71 429 857 (17.4)	0.311
Diseases of the respiratory system	34 492 570 (8.3)	233 820 (3.8)	34 258 750 (8.4)	0.166
Diseases of the skin and subcutaneous tissue	7 419 371 (1.8)	217 315 (3.5)	7 202 057 (1.8)	0.133
Endocrine, nutritional, and metabolic diseases	16 014 737 (3.8)	175 525 (2.8)	15 839 212 (3.9)	0.054
Diseases of the digestive system	38 827 784 (9.3)	171 090 (2.8)	38 656 694 (9.4)	0.229
Diseases of the musculoskeletal system and connective tissue	19 337 003 (4.6)	133 365 (2.2)	19 203 639 (4.7)	0.120
Diseases of the nervous system	12 094 530 (2.9)	118 090 (1.9)	11 976 440 (2.9)	0.060
Diseases of the genitourinary system	16 979 533 (4.1)	85 495 (1.4)	16 894 038 (4.1)	0.138
Pregnancy, childbirth, and puerperium	29 850 763 (7.2)	73 965 (1.2)	29 776 798 (7.3)	0.235
Neoplasms	21 827 586 (5.2)	67 715 (1.1)	21 759 871 (5.3)	0.189
Symptoms, signs, and abnormal clinical laboratory findings, not elsewhere classified	8 467 245 (2.0)	65 830 (1.1)	8 401 415 (2.0)	0.070
Diseases of the blood and blood-forming organs and certain disorders involving the immune mechanism	4 748 999 (1.1)	21 430 (0.3)	4 727 569 (1.2)	0.076
Factors influencing health status and contact with health services	6 032 843 (1.4)	18 940 (0.3)	6 013 903 (1.5)	0.097
Diseases of the eye and adnexa	234 540 (0.1)	6645 (0.1)	227 895 (0.1)	0.022
Congenital malformations, deformations, and chromosomal abnormalities	551 060 (0.1)	1925 (<0.01)	549 135 (0.1)	0.028
Diseases of the ear and mastoid process	262 290 (0.1)	1520 (<0.01)	260 770 (0.1)	0.016
External causes of morbidity	1525 (<0.01)	20 (<0.01)	1505 (<0.01)	0.000
Certain conditions originating in the perinatal period^c^	3695 (<0.01)	0	3695 (<0.01)	NA
Total inpatient	416 199 050 (100)	6 197 196 (1.5)	410 001 857 (98.5)	NA
Mean length of stay (95% CI), d per admission	4.8 (4.7-4.8)	6.7 (6.5-6.7)	4.8 (4.7-4.8)	0.284

Abbreviation: NA, not applicable.

^a^ Data source: National Inpatient Sample, 2017 to 2019.

^b^ Standardized difference displays the absolute value of the difference in proportions divided by the standard error and is an indicator of effect size (Cohen *d*) (>0.20 indicates small; >0.50, medium; and ≥.80, large effect sizes).

^c^ Certain conditions originating in the perinatal period had no coded housing instability so no standardized difference is reported.

Costs of hospitalizations with and without coded housing instability by diagnoses are displayed in Table 4. Admissions with coded housing instability accounted for $9.3 billion (0.8%) of the total $1.2 trillion cost of admissions between 2017 and 2019. Of that $9.3 billion, $3.5 billion (37.9%) was attributed to the 475 575 admissions with both coded housing instability and mental, behavioral, and neurodevelopmental disorders (vs $31.7 billion [2.7%] without coded housing instability). Diseases of the circulatory system accounted for the greatest percentage of costs for admissions without coded housing instability ($254.7 billion [21.8%]). Admissions for injury, poisoning, and certain other consequences of external causes accounted for the second highest costs of admissions among patients with coded housing instability ($1.1 billion [11.7%]).

**Table 4.** Cost of Hospitalization by Diagnosis Among Patients With and Without Coded Housing Instability^a^

Discharge diagnosis	Cost in millions, $ (%)			Standardized difference (Cohen *d*)^b^
	Total	With coded housing instability	Without coded housing instability	
Mental, behavioral, and neurodevelopmental disorders	35 276.3 (3.0)	3531.5 (37.9)	31 744.8 (2.7)	2.076
Injury, poisoning, and certain other consequences of external causes	139 294.5 (11.8)	1090.9 (11.7)	138 203.5 (11.9)	0.004
Diseases of the circulatory system	255 556.2 (21.7)	878.4 (9.4)	254 677.8 (21.8)	0.301
Certain infections and parasitic diseases	127 900.8 (10.9)	860.9 (9.2)	127 040.0 (10.9)	0.053
Diseases of the respiratory system	82 281.5 (7.0)	522.8 (5.6)	81 758.7 (7.0)	0.055
Diseases of the skin and subcutaneous tissue	14 197.5 (1.2)	432.2 (4.6)	13 765.3 (1.2)	0.316
Diseases of the digestive system	105 878.5 (9.0)	416.6 (4.5)	105 462.0 (9.0)	0.160
Endocrine, nutritional, and metabolic diseases	42 623.5 (3.6)	360.0 (3.9)	42 263.5 (3.6)	0.013
Diseases of the musculoskeletal system and connective tissue	114 480.0 (9.7)	294.1 (3.2)	114 185.9 (9.8)	0.224
Diseases of the nervous system	28 464.5 (2.4)	220.8 (2.4)	28 243.7 (2.4)	0.003
Neoplasms	79 219.7 (6.7)	163.6 (1.8)	79 056.1 (6.8)	0.201
Diseases of the genitourinary system	38 542.7 (3.3)	162.1 (1.7)	38 380.6 (3.3)	0.087
Symptoms, signs, and abnormal clinical laboratory findings, not elsewhere classified	20 327.0 (1.7)	145.4 (1.6)	20 181.6 (1.7)	0.013
Pregnancy, childbirth, and puerperium	60 648.8 (5.2)	131.0 (1.4)	60 517.8 (5.2)	0.171
Diseases of the blood and blood-forming organs and certain disorders involving the immune mechanism	12 014.9 (1.0)	49.3 (0.5)	11 965.6 (1.0)	0.049
Factors influencing health status and contact with health services	14 123.3 (1.2)	30.7 (0.3)	14 092.6 (1.2)	0.081
Congenital malformations, deformations, and chromosomal abnormalities	3351.7 (0.3)	6.2 (0.1)	3345.5 (0.3)	0.041
Diseases of the eye and adnexa	636.9 (0.1)	13.9 (0.1)	623.0 (0.1)	0.041
Certain conditions originating in the perinatal period^c^	10.9 (<0.01)	0.0	10.9 (<0.01)	NA
Diseases of the ear and mastoid process	702.0 (0.1)	4.1 (<0.01)	697.9 (0.1)	0.007
External causes of morbidity	4.8 (<0.01)	0.0	4.7 (<0.01)	0.000
Total	1 175 536.0 (100)	9314.5 (0.8)	1 166 221.5 (99.2)	NA

Abbreviation: NA, not applicable.

^a^ Data source: National Inpatient Sample, 2017 to 2019.

^b^ “Standardized difference” displays the absolute value of the difference in proportions divided by the standard error and is an indicator of effect size (Cohen *d*) (>0.20 indicates small; >0.50,medium; and ≥.80, large effect sizes).

^c^ “Certain conditions originating in the perinatal period” had no coded housing instability so no standardized difference is reported.

Sensitivity analyses were performed to examine differences between hospitalizations with and without each of the 5 Z59-codes used in the study. General trends were consistent with original comparisons of patient characteristics (eTable 3 in the Supplement), admissions (eTables 4-6 in the Supplement), length of stay (eTables 7 and 8 in the Supplement), and cost (eTables 9 and 10 in the Supplement) by housing instability. When comparing hospitalizations with and without each of the 5 housing instability Z59-codes by reason for admission, large effect sizes were found for differences in admission rates (eTables 4-6 in the Supplement), length of stay (eTables 7 and 8 in the Supplement), and costs (eTables 9 and 10 in the Supplement) among patients with mental, behavioral, and neurodevelopmental disorders.

## Discussion

In this cross-sectional study that compared common primary diagnoses, length of stay, and costs among hospitalized patients with and without coded housing instability between 2017 and 2019, 3 principal findings were observed. First, the most common reason for hospitalization among patients with coded housing instability was mental, behavioral, and neurodevelopmental disorders. This diagnosis accounted for 50.3% of hospitalizations and nearly 60% of inpatient days for patients with coded housing instability. Second, hospitalized patients with coded housing instability had a higher mean length of stay compared with patients without coded housing instability (6.7 vs 4.8 days). Third, hospitalization costs for patients with coded housing instability were $9.3 billion, with 37.9% of those costs from primary diagnoses of mental, behavioral, and neurodevelopmental disorders. Taken together, findings suggest that future attention to understanding the association between housing instability and inpatient admissions for mental, behavioral, and neurodevelopmental disorders is warranted.

This study adds to the existing literature on housing instability and health via analysis of 3 years of nationally representative health outcome data. Prior work^23^ largely relied on nonrepresentative or self-reported data. Findings from this study were consistent with prior work that documented higher prevalence of mental and behavioral health disorders, hospitalizations, and health care costs among people experiencing housing instability, and especially homelessness.^4,30,31^

By including multiple levels of housing instability in addition to homelessness, results suggested that trends in diagnoses, utilization, and costs may extend across the housing instability continuum. Much of the existing literature exclusively examined homelessness, which exacerbates mental health and substance use conditions due to a lack of safety, inability to adhere to treatment and manage chronic conditions, and a lack of or disrupted care that can lead to increased anxiety, depression, paranoia, and avoidable hospitalization.^4^ Other aspects of housing instability captured by the 5 Z59-codes and examined via sensitivity analyses highlight the potential relevance of the complete housing instability continuum when examining hospitalizations, length of stay, and costs by reason for admission. Results also supported the need for more sensitive and widely used housing instability measures. This study also adds to prior work^32,33,34,35,36,37,38^ quantifying health care costs associated with housing instability and homelessness by using nationally representative hospitalization data. People experiencing housing instability, homelessness, and poverty face numerous health risks and use costly hospital-based acute care at disproportionately higher rates.^39^ This population is among the top 5% of hospital users estimated to account for 50% of health care costs.^32,39^ By providing an unadjusted national estimate of inpatient hospitalization costs among patients with coded housing instability, study findings provide baseline information to build on work making a clear business case for health systems and payers to address SDOH.^39^

Our study findings have implications for a range of stakeholders addressing housing and social needs to improve health care delivery.^1^ First, the association between coded housing instability and higher rates of admissions, inpatient days, and costs has potential implications for payers and health systems. Housing and other SDOH will become increasingly important to promote health as payers transition from volume to value-based payment models^32^ and Medicare and Medicaid implement strategies focused on SDOH.^40^ Prior studies document how housing can improve health care effectiveness for patients, particularly for beneficiaries of Medicaid and similar programs.^2^ Specifically, health systems may find a practical business case to invest in affordable housing and supportive social services as a strategy to reduce inpatient hospital utilization associated with housing instability.^1,39^ Similarly, health systems and payers may consider other strategies, such as advocating for inclusionary zoning ordinances to increase the availability of affordable multifamily dwellings for renters at high risk of housing instability.^31^

Second, the high proportion of admissions for mental, behavioral, and neurodevelopmental disorders among patients with coded housing instability suggests that research and efforts addressing the intersection of housing instability, mental and behavioral health services, and inpatient health care utilization are likely areas of high impact. Specifically, efforts across multiple sectors may find mutually beneficial areas of interest. Findings from the present study further suggest that health care programs designed to address the needs of patients experiencing homelessness may also benefit other patients across the housing instability continuum. Third, findings support efforts to improve screening instruments and documentation methods. The increasing focus on housing instability and SDOH by clinicians and payers will require standardized, widely adopted protocols to develop interventions and evaluate effectiveness. This study generated baseline associations to inform future work in this area.

### Limitations

Results of this cross-sectional study must be interpreted within the context of its limitations. First, because this study focused on adults aged 18 years and older, correlational findings may not extend to the younger US population. The NIS, however, is the largest publicly available all-payer data set in the US and therefore provides the most generalizable outcome estimates for adults. Second, large claims data sets including the NIS do not provide the clinical granularity of health records. To minimize effects of this limitation, only reliably identifiable outcomes in the NIS were used (primary diagnoses, inpatient days, and cost).

Third, cross-sectional study results cannot be interpreted causally. The unadjusted comparisons did not address potential confounders, such as differential use of Z-codes by reason for admission. More representative data collected via consistent and standardized screening are required to fully understand the association between housing instability and reason for hospitalization. Nevertheless, the descriptive comparisons produced by this study using a nationally representative data set provide critical baseline information. This information is necessary to improve social risk documentation and develop interventions focused on the association between housing instability and inpatient health care utilization, especially among patients admitted for mental, behavioral, and neurodevelopmental disorders.

Fourth, housing instability definitions widely vary and no standardized measure exists. This study relied on 5 imperfect and underutilized *ICD-10* Z59-codes that, according to the literature, are likely more specific than sensitive indicators of housing instability.^41,42,43,44,45^ The Z59-codes likely capture the most extreme experience of housing instability: homelessness. Studies^26,41,43,46,47,48,49^ have also shown that these and other SDOH-related Z-codes (Z55-65) inconsistently map to SDOH screening instruments. Moreover, study results were consistent with prior work^25,42,43,44^ that demonstrated that Z-codes Z55-65 are only found in 1% to 2% of inpatient medical records. An additional study^16^ found that SDOH, including housing instability, were underrepresented in administrative data compared with physician notes. This prior work further supports that results of the present study reveal the “tip of the iceberg.”

Despite these limitations, *ICD-10* Z-codes remain an important metric for measuring health disparities. They are already being used ubiquitously in clinical practice across US hospitals and internationally.^50^ *ICD-10* codes are used by all World Health Organization (WHO) member nations, translated into 43 languages, and serve as the WHO’s basis for reporting health status, mortality, and medical reimbursements.

## Conclusions

This cross-sectional analysis of a nationally representative data set found that coded housing instability among hospitalized patients was associated with higher rates of inpatient admissions for mental, behavioral, and neurodevelopmental disorders, longer hospital stays, and substantial health care costs. This association, however, is likely the tip of the iceberg and requires attention and cooperation from multiple sectors to improve screening, deepen understanding of the housing and health care utilization linkage by diagnosis, and develop effective interventions. These findings suggest that multisector efforts aimed at improving housing instability, mental and behavioral health services, and efficient utilization of inpatient hospital services may find opportunities for synergistic collaboration.

## Notes

Supplement. **eTable 1.** *ICD-10* Codes Used to Create Primary Discharge Diagnoses Categories **eTable 2.** NIS Sample Details **eTable 3.** Characteristics of Hospitalized Patients Without and With Each of 5 Housing Instability Z59-Codes **eTable 4.** Number of Hospital Admissions by Diagnosis Among Patients Without and With Housing Instability Codes Z59.0 (Homelessness) and Z59.1 (Inadequate Housing) **eTable 5.** Number of Hospital Admissions by Diagnosis Among Patients Without and With Housing Instability Codes Z59.2 (Discord With Neighbors, Lodgers, and Landlords) and Z59.3 (Problems Related to Living in a Residential Institution) **eTable 6.** Number of Hospital Admissions by Diagnosis Among Patients Without and With Housing Instability Code Z59.8 (Other Housing and Economic Problems) **eTable 7.** Total Inpatient Days and Mean Length of Stay by Diagnosis Among Patients With and Without Housing Instability Codes Z59.0 (Homelessness), Z59.1 (Inadequate Housing), and Z59.2 (Discord With Neighbors, Lodgers, and Landlords) **eTable 8.** Total Inpatient Days and Mean Length of Stay by Diagnosis Among Patients With and Without Housing Instability Codes Z59.3 (Problems Related to Living in a Residential Institution) and Z59.8 (Other Housing and Economic Problems) **eTable 9.** Cost (Millions, USD) of Hospitalization by Diagnosis Among Patients With and Without Housing Instability Codes Z59.0 (Homelessness), Z59.1 (Inadequate Housing), and Z59.2 (Discord With Neighbors, Lodgers, and Landlords) **eTable 10.** Cost (Millions, USD) of Hospitalization by Diagnosis Among Patients With and Without Housing Instability Codes Z59.3 (Problems Related to Living in a Residential Institution) and Z59.8 (Other Housing and Economic Problems) **eFigure.** Most Common Reasons for Hospitalization Among Patients With and Without Coded Housing Instability
