# Supplementary material for: Association of Coded Housing Instability and Hospitalization in the US
Source: JAMA Netw Open. 2022 Nov 14;5(11):e2241951. doi: 10.1001/jamanetworkopen.2022.41951 (PMC9664259; doi:10.1001/jamanetworkopen.2022.41951)
Supplement: Supplement. — eTable 1. ICD-10 Codes Used to Create Primary Discharge Diagnoses Categories eTable 2. NIS Sample Details eTable 3. Characteristics of Hospitalized Patients Without and With Each of 5 Housing Instability Z59-Codes eTable 4. Number of Hospital Admissions by Diagnosis Among Patients Without and With Housing Instability Codes Z59.0 (Homelessness) and Z59.1 (Inadequate Housing) eTable 5. Number of Hospital Admissions by Diagnosis Among Patients Without and With Housing Instability Codes Z59.2 (Discord With Neighbors, Lodgers, and Landlords) and Z59.3 (Problems Related to Living in a Residential Institution) eTable 6. Number of Hospital Admissions by Diagnosis Among Patients Without and With Housing Instability Code Z59.8 (Other Housing and Economic Problems) eTable 7. Total Inpatient Days and Mean Length of Stay by Diagnosis Among Patients With and Without Housing Instability Codes Z59.0 (Homelessness), Z59.1 (Inadequate Housing), and Z59.2 (Discord With Neighbors, Lodgers, and Landlords) eTable 8. Total Inpatient Days and Mean Length of Stay by Diagnosis Among Patients With and Without Housing Instability Codes Z59.3 (Problems Related to Living in a Residential Institution) and Z59.8 (Other Housing and Economic Problems) eTable 9. Cost (Millions, USD) of Hospitalization by Diagnosis Among Patients With and Without Housing Instability Codes Z59.0 (Homelessness), Z59.1 (Inadequate Housing), and Z59.2 (Discord With Neighbors, Lodgers, and Landlords) eTable 10. Cost (Millions, USD) of Hospitalization by Diagnosis Among Patients With and Without Housing Instability Codes Z59.3 (Problems Related to Living in a Residential Institution) and Z59.8 (Other Housing and Economic Problems) eFigure. Most Common Reasons for Hospitalization Among Patients With and Without Coded Housing Instability [file jamanetwopen-e2241951-s001.pdf]

Supplemental Online Content

Rollings KA, Kunnath N, Ryus CR, Janke AT, Ibrahim AM. Association of coded housing instability and hospitalization in the US. *JAMA Netw Open*. 2022;5(11):e2241951. doi:10.1001/jamanetworkopen.2022.41951

eTable 1. *ICD-10* Codes Used to Create Primary Discharge Diagnoses Categories
eTable 2. NIS Sample Details
eTable 3. Characteristics of Hospitalized Patients Without and With Each of 5 Housing Instability Z59-Codes
eTable 4. Number of Hospital Admissions by Diagnosis Among Patients Without and With Housing Instability Codes Z59.0 (Homelessness) and Z59.1 (Inadequate Housing)
eTable 5. Number of Hospital Admissions by Diagnosis Among Patients Without and With Housing Instability Codes Z59.2 (Discord With Neighbors, Lodgers, and Landlords) and Z59.3 (Problems Related to Living in a Residential Institution)
eTable 6. Number of Hospital Admissions by Diagnosis Among Patients Without and With Housing Instability Code Z59.8 (Other Housing and Economic Problems)
eTable 7. Total Inpatient Days and Mean Length of Stay by Diagnosis Among Patients With and Without Housing Instability Codes Z59.0 (Homelessness), Z59.1 (Inadequate Housing), and Z59.2 (Discord With Neighbors, Lodgers, and Landlords)
eTable 8. Total Inpatient Days and Mean Length of Stay by Diagnosis Among Patients With and Without Housing Instability Codes Z59.3 (Problems Related to Living in a Residential Institution) and Z59.8 (Other Housing and Economic Problems)
eTable 9. Cost (Millions, USD) of Hospitalization by Diagnosis Among Patients With and Without Housing Instability Codes Z59.0 (Homelessness), Z59.1 (Inadequate Housing), and Z59.2 (Discord With Neighbors, Lodgers, and Landlords)
eTable 10. Cost (Millions, USD) of Hospitalization by Diagnosis Among Patients With and Without Housing Instability Codes Z59.3 (Problems Related to Living in a Residential Institution) and Z59.8 (Other Housing and Economic Problems)
eFigure. Most Common Reasons for Hospitalization Among Patients With and Without Coded Housing Instability

This supplemental material has been provided by the authors to give readers additional information about their work.

© 2022 Rollings KA et al. *JAMA Network Open.*

eTable 1. *ICD-10* Codes Used to Create Primary Discharge Diagnoses Categories	
**ICD-10 Codes**	**Category**
A00-B99	Certain infections and parasitic diseases
C00-D49	Neoplasms
D50-D89	Diseases of the blood/blood-forming organs and certain disorders involving the immune
E00-E89	Endocrine, nutritional and metabolic diseases
F01-F99	Mental, Behavioral and Neurodevelopmental disorders
G00-G99	Diseases of the nervous system
H00-H59	Diseases of the eye and adnexa
H60-H95	Diseases of the ear and mastoid process
I00-I99	Diseases of the circulatory system
J00-J99	Diseases of the respiratory system
K00-K95	Diseases of the digestive system
L00-L99	Diseases of the skin and subcutaneous tissue
M00-M99	Diseases of the musculoskeletal system and connective tissue
N00-N99	Diseases of the genitourinary system
O00-O9A	Pregnancy, childbirth, and puerperium
P00-P96	Certain conditions originating in the perinatal period
Q00-Q99	Congenital malformations, deformations and chromosomal abnormalities
R00-R99	Symptoms, signs, and abnormal clinical laboratory findings, not elsewhere classified
S00-T88	Injury, poisoning, and certain other consequences of external causes
V00-Y99	External causes of morbidity
Z00-Z99	Factors influencing health status and contact with health services

Data source: International Classification of Diseases, Tenth Revision, Clinical Modification (ICD-10-CM)

© 2022 Rollings KA et al. *JAMA Network Open.*

**eTable 2. NIS Sample Details**				
**Inclusion/Exclusion Criteria**	**Total**	**NIS year**		
		**2017**	**2018**	**2019**
Total NIS sample (with survey weights)	106,744,957	35,798,453	35,527,481	35,419,023
Excluded:				
<18 or >99 years of age	15,845,919	5,377,546	5,267,618	5,200,755
Missing study data	3,550,437	1,425,260	1,079,620	1,045,557
Total N	87,348,601	28,995,647	29,180,243	29,172,711

Data source: National Inpatient Sample (NIS), 2017-2019

© 2022 Rollings KA et al. *JAMA Network Open.*

eTable 3. Characteristics of Hospitalized Patients Without and With Each of Five Housing Instability Z59-Codes											
	**Without Coded Housing Instability**	**With Coded Housing Instability Z59-code**[a]									
**Characteristic**	admissions (%) (N=86,403,514)	**Z59.0** admissions (%) (N=915,305)	**Stand. Diff.**[b]	**Z59.1** admissions (%) (N=5,030)	**Stand. Diff.**[b]	**Z59.2** admissions (%) (N=1,220)	**Stand. Diff.**[b]	**Z59.3** admissions (%) (N=3,335)	**Stand. Diff.**[b]	**Z59.8** admissions (%) (N=24,445)	**Stand. Diff.**[b]
**Age**, mean years (SD)	58.4 (20.2)	45.4 (13.8)	0.647	51.3 (20.3)	0.355	43.6 (16.3)	0.736	68.6 (19.6)	0.504	44 (15.9)	0.715
**Sex**											
Male	36,506,229 (42.3)	653430 (71.4)	0.590	2640 (52.5)	0.207	650 (53.3)	0.223	1485 (44.5)	0.046	12980 (53.1)	0.220
Female	49,897,282 (57.7)	261875 (28.6)	(ref)	2390 (47.5)	(ref)	570 (46.7)	(ref)	1850 (55.5)	(ref)	11465 (46.9)	(ref)
**Race**											
Asian or Pacific Islander	2,429,708 (2.8)	11440 (1.2)	0.095	40 (0.8)	0.122	5 (0.4)	0.145	145 (4.3)	0.093	330 (1.3)	0.088
Black	12,929,158 (15.0)	229425 (25.1)	0.282	1300 (25.8)	0.305	365 (29.9)	0.419	470 (14.1)	0.024	5050 (20.7)	0.160
Hispanic	9,697,867 (11.2)	105360 (11.5)	0.009	500 (9.9)	0.041	70 (5.7)	0.174	330 (9.9)	0.042	2290 (9.4)	0.059
Native American	553,120 (0.6)	12655 (1.4)	0.093	80 (1.6)	0.119	0 (0)	0.080	0 (0)	0.080	215 (0.9)	0.030
White	58,218,489 (67.4)	525130 (57.4)	0.213	2985 (59.3)	0.171	750 (61.5)	0.126	2245 (67.3)	0.001	15950 (65.2)	0.045
Other[c]	2,575,169 (3.0)	31295 (3.4)	0.026	125 (2.5)	0.029	30 (2.5)	0.031	145 (4.3)	0.080	610 (2.5)	0.029
**Insurance Type**											
Private	22,999,849 (26.6)	66480 (7.3)	0.439	665 (13.2)	0.303	195 (16)	0.241	260 (7.8)	0.426	5130 (21)	0.127
Medicare	41,976,918 (48.6)	189370 (20.7)	0.559	1825 (36.3)	0.246	460 (37.7)	0.218	2480 (74.4)	0.516	4575 (18.7)	0.598
Medicaid	15,541,175 (18.0)	511430 (55.9)	0.983	1980 (39.4)	0.557	455 (37.3)	0.503	495 (14.8)	0.082	9090 (37.2)	0.500
Other[c]	2,408,729 (2.8)	35050 (3.8)	0.063	150 (3)	0.012	10 (0.8)	0.120	45 (1.3)	0.087	795 (3.3)	0.028
Uninsured	3,476,841 (4.0)	112975 (12.3)	0.419	410 (8.2)	0.210	100 (8.2)	0.212	55 (1.6)	0.121	4855 (19.9)	0.806
**Elective Procedure**	19,786,680 (22.9)	66080 (7.2)	0.374	860 (17.1)	0.138	160 (13.1)	0.233	310 (9.3)	0.324	2145 (8.8)	0.336
**Emergency Admittance**	53,325,351 (61.7)	690830 (75.5)	0.283	3245 (64.5)	0.058	855 (70.1)	0.172	2575 (77.2)	0.319	15390 (63)	0.026
**Major Operating Room Procedure**	9,179,722 (31.8)	26405 (7.9)	0.516	190 (10.8)	0.452	5 (0.7)	0.669	90 (7.3)	0.527	620 (7.2)	0.530
**Discharge Description**											
Routine	18,060,035 (62.7)	694380 (75.9)	0.270	3000 (59.6)	0.069	1030 (84.4)	0.467	890 (26.7)	0.617	20765 (84.9)	0.456
Transfer to Short Term Hospital	580,429 (2.0)	16200 (1.8)	0.019	130 (2.6)	0.018	20 (1.6)	0.143	85 (2.5)	0.058	325 (1.3)	0.082
Other Transfer	4,736,515 (16.4)	112495 (12.3)	0.116	1245 (24.8)	0.138	110 (9)	0.154	2100 (63)	1.152	1910 (7.8)	0.238
Home Health Care	4,344,196 (15.1)	14500 (1.6)	0.377	405 (8.1)	0.247	30 (2.5)	0.346	145 (4.3)	0.319	805 (3.3)	0.321
Against Medical Advice	451,855 (1.6)	75325 (8.2)	0.548	220 (4.4)	0.170	30 (2.5)	0.018	40 (1.2)	0.094	595 (2.4)	0.111

Data source: National Inpatient Sample (NIS), 2017-2019

[a] = *Z59.0= homelessness; Z59.1= inadequate housing; Z59.2= discord with neighbors, lodgers, landlords; Z59.3= problems related to living in residential institution; Z59.8= other.*

[b] = *"Standardized difference" displays the absolute value of the difference in proportions divided by the standard error and is an indicator of effect size (Cohen d) (0.20-0.49 indicates small; 0.50 to 0.79 medium; and ≥0.80, large effect sizes)."*

[c] = *"Other" race includes multiple races and state-reported race categories excluded from other NIS categories.*

© 2022 Rollings KA et al. *JAMA Network Open.*

eTable 4. Number of Hospital Admissions by Diagnosis Among Patients Without and With Housing Instability Codes Z59.0 (Homelessness) and Z59.1 (Inadequate Housing)

**Discharge Diagnosis**	**Without Coded Housing Instability** admissions (%)	**With Coded Housing Instability Z59-code**[a]					
		**Z59.0** admissions (%)	**Odds Ratio** (95% CI)	**Stand. Diff.**[b]	**Z59.1** admissions (%)	**Odds Ratio** (95% CI)	**Stand. Diff.**[b]
Mental, behavioral & neurodevelopmental disorders	4,470,675 (5.2)	460035 (50.3)	18.52 (17.81-19.25)	1.993	2140 (42.5)	13.57 (10.16-18.12)	1.687
Injury, poisoning, & certain other consequences of external causes	7,559,439 (8.7)	67560 (7.4)	0.83 (0.8-0.86)	0.048	355 (7.1)	0.79 (0.6-1.05)	0.060
Diseases of the circulatory system	14,452,895 (16.7)	62095 (6.8)	0.36 (0.35-0.37)	0.267	390 (7.8)	0.42 (0.32-0.55)	0.240
Certain infections & parasitic diseases	7,128,154 (8.2)	55000 (6.0)	0.71 (0.68-0.74)	0.082	190 (3.8)	0.44 (0.31-0.62)	0.163
Diseases of the respiratory system	6,918,933 (8.0)	49665 (5.4)	0.66 (0.64-0.68)	0.095	355 (7.1)	0.87 (0.64-1.19)	0.035
Diseases of the skin & subcutaneous tissue	1,605,849 (1.9)	46195 (5.0)	2.81 (2.7-2.92)	0.234	180 (3.6)	1.96 (1.35-2.84)	0.127
Endocrine, nutritional & metabolic diseases	3,761,624 (4.4)	37080 (4.1)	0.93 (0.9-0.96)	0.015	205 (4.1)	0.93 (0.66-1.32)	0.014
Diseases of the digestive system	8,581,113 (9.9)	36335 (4.0)	0.37 (0.36-0.39)	0.200	120 (2.4)	0.22 (0.14-0.34)	0.252
Diseases of the musculoskeletal system & connective tissue	6,240,469 (7.2)	18940 (2.1)	0.27 (0.26-0.28)	0.200	90 (1.8)	0.23 (0.14-0.38)	0.210
Diseases of the genitourinary system	4,085,434 (4.7)	17390 (1.9)	0.39 (0.37-0.41)	0.134	170 (3.4)	0.7 (0.48-1.04)	0.064
Symptoms, signs, & abnormal clinical laboratory findings, not elsewhere classified	2,401,400 (2.8)	17350 (1.9)	0.68 (0.65-0.71)	0.054	165 (3.3)	1.19 (0.81-1.74)	0.030
Diseases of the nervous system	2,226,485 (2.6)	16625 (1.8)	0.7 (0.67-0.73)	0.048	110 (2.2)	0.85 (0.55-1.3)	0.025
Pregnancy, childbirth, & puerperium	11,157,270 (12.9)	16350 (1.8)	0.12 (0.12-0.13)	0.333	450 (8.9)	0.66 (0.49-0.9)	0.118
Neoplasms	3,559,619 (4.1)	6790 (0.7)	0.17 (0.16-0.19)	0.171	45 (0.9)	0.21 (0.11-0.41)	0.162
Diseases of the blood & blood-forming organs & certain disorders involving the immune mechanism	1,074,870 (1.2)	4185 (0.5)	0.36 (0.34-0.39)	0.071	30 (0.6)	0.48 (0.21-1.08)	0.058
Factors influencing health status & contact with health services	908,655 (1.1)	2250 (0.2)	0.23 (0.2-0.26)	0.079	35 (0.7)	0.66 (0.31-1.39)	0.035
Diseases of the eye & adnexa[c]	65,820 (0.1)	885 (0.1)	1.27 (1.09-1.48)	0.007	0.0 (0.0)	NA	NA
Certain conditions originating in the perinatal period[c]	905 (0.0)	0 (0.0)	NA	NA	0.0 (0.0)	NA	NA
Congenital malformations, deformations & chromosomal abnormalities[c]	109,530 (0.1)	220 (0.0)	0.19 (0.14-0.26)	0.029	0.0 (0.0)	NA	NA
Diseases of the ear & mastoid process[c]	93,980 (0.1)	350 (0.0)	0.35 (0.28-0.44)	0.021	0.0 (0.0)	NA	NA
External causes of morbidity[c]	395 (0.0)	5 (0.0)	1.19 (0.18-7.79)	<0.001	0.0 (0.0)	NA	NA
**Total number of hospital admissions**	**86,403,514** (98.9)	**915,305** (96.8)	NA	NA	**5,030** (0.5)	NA	NA

Data source: Nationwide Inpatient Sample (NIS), 2017-2019

Abbreviations: NA, not applicable.

[a] = *Z59.0= homelessness; Z59.1= inadequate housing; Z59.2= discord with neighbors, lodgers, landlords; Z59.3= problems related to living in residential institution; Z59.8= other.*

[b] = *"Standardized difference" displays the absolute value of the difference in proportions divided by the standard error and is an indicator of effect size (Cohen d) (0.20-0.49 indicates small; 0.50 to 0.79 medium; and ≥0.80, large effect sizes)."*

[c] = *No standardized difference is reported for a hospitalization discharge diagnosis that had none of the particular housing instability Z59-code.*

© 2022 Rollings KA et al. *JAMA Network Open.*

eTable 5. Number of Hospital Admissions by Diagnosis Among Patients Without and With Housing Instability Codes Z59.2 (Discord With Neighbors, Lodgers, and Landlords) and Z59.3 (Problems Related to Living in a Residential Institution)

**Discharge Diagnosis**	**Without Coded Housing Instability** admissions (%)	**With Coded Housing Instability Z59-code**[a]					
		**Z59.2** admissions (%)	**Odds Ratio** (95% CI)	**Stand. Diff.**[b]	**Z59.3** admissions (%)	**Odds Ratio** (95% CI)	**Stand. Diff.**[b]
Mental, behavioral & neurodevelopmental disorders	4,470,675 (5.2)	1070 (87.7)	130.73 (86.19-198.28)	3.726	915 (27.4)	6.93 (4.21-11.41)	1.005
Injury, poisoning, & certain other consequences of external causes	7,559,439 (8.7)	35 (2.9)	0.31 (0.13-0.7)	0.208	250 (7.5)	0.85 (0.62-1.16)	0.044
Diseases of the circulatory system	14,452,895 (16.7)	15 (1.2)	0.06 (0.02-0.2)	0.415	320 (9.6)	0.53 (0.39-0.71)	0.191
Certain infections & parasitic diseases	7,128,154 (8.2)	15 (1.2)	0.14 (0.04-0.45)	0.255	425 (12.7)	1.62 (1.15-2.29)	0.163
Diseases of the respiratory system	6,918,933 (8.0)	5 (0.4)	0.05 (0.01-0.35)	0.280	460 (13.8)	1.84 (1.42-2.38)	0.213
Diseases of the skin & subcutaneous tissue	1,605,849 (1.9)	0.0 (0.0)	-	0.138	30 (0.9)	0.48 (0.22-1.04)	0.071
Endocrine, nutritional & metabolic diseases	3,761,624 (4.4)	15 (1.2)	0.27 (0.08-0.91)	0.153	115 (3.4)	0.78 (0.51-1.21)	0.044
Diseases of the digestive system	8,581,113 (9.9)	10 (0.8)	0.07 (0.02-0.32)	0.305	240 (7.2)	0.7 (0.52-0.95)	0.091
Diseases of the musculoskeletal system & connective tissue	6,240,469 (7.2)	5 (0.4)	0.05 (0.01-0.39)	0.263	30 (0.9)	0.12 (0.05-0.28)	0.244
Diseases of the genitourinary system	4,085,434 (4.7)	15 (1.2)	0.25 (0.08-0.83)	0.165	210 (6.3)	1.35 (0.99-1.84)	0.074
Symptoms, signs, & abnormal clinical laboratory findings, not elsewhere classified	2,401,400 (2.8)	10 (0.8)	0.29 (0.07-1.22)	0.119	80 (2.4)	0.86 (0.5-1.47)	0.023
Diseases of the nervous system	2,226,485 (2.6)	20 (1.6)	0.63 (0.18-2.19)	0.059	170 (5.1)	2.03 (1.33-3.1)	0.159
Pregnancy, childbirth, & puerperium	11,157,270 (12.9)	5 (0.4)	0.03 (0-0.2)	0.373	10 (0.3)	0.02 (0-0.08)	0.376
Neoplasms[c]	3,559,619 (4.1)	0.0 (0.0)	NA	NA	20 (0.6)	0.14 (0.06-0.35)	0.177
Diseases of the blood & blood-forming organs & certain disorders involving the immune mechanism[c]	1,074,870 (1.2)	0.0 (0.0)	NA	NA	30 (0.9)	0.72 (0.33-1.6)	0.031
Factors influencing health status & contact with health services[c]	908,655 (1.1)	0.0 (0.0)	NA	NA	20 (0.6)	0.57 (0.19-1.7)	0.044
Diseases of the eye & adnexa[c]	65,820 (0.1)	0.0 (0.0)	NA	NA	5 (0.1)	1.97 (0.27-14.19)	0.027
Certain conditions originating in the perinatal period[c]	905 (0.0)	0.0 (0.0)	NA	NA	0.0 (0.0)	NA	NA
Congenital malformations, deformations & chromosomal abnormalities[c]	109,530 (0.1)	0.0 (0.0)	NA	NA	0.0 (0.0)	NA	NA
Diseases of the ear & mastoid process[c]	93,980 (0.1)	0.0 (0.0)	NA	NA	5 (0.1)	1.38 (0.21-9.14)	0.012
External causes of morbidity[c]	395 (0.0)	0.0 (0.0)	NA	NA	0.0 (0.0)	NA	NA
**Total number of hospital admissions**	**86,403,514** (98.9)	**1,220** (0.1)	NA	NA	**3,335** (0.4)	NA	NA

Data source: Nationwide Inpatient Sample (NIS), 2017-2019

Abbreviations: NA, not applicable.

[a] = *Z59.0= homelessness; Z59.1= inadequate housing; Z59.2= discord with neighbors, lodgers, landlords; Z59.3= problems related to living in residential institution; Z59.8= other.*

[b] = *"Standardized difference" displays the absolute value of the difference in proportions divided by the standard error and is an indicator of effect size (Cohen d) (0.20-0.49 indicates small; 0.50 to 0.79 medium; and ≥0.80, large effect sizes)."*

[c] = *No standardized difference is reported for a hospitalization discharge diagnosis that had none of the particular housing instability Z59-code.*

© 2022 Rollings KA et al. *JAMA Network Open.*

eTable 6. Number of Hospital Admissions by Diagnosis Among Patients Without and With Housing Instability Code Z59.8 (Other Housing and Economic Problems)

Discharge Diagnosis	Without Coded Housing Instability admissions (%)	With Coded Housing Instability Z59-code[a]		
		Z59.8 admissions (%)	Odds Ratio (95% CI)	Stand. Diff.[b]
Mental, behavioral & neurodevelopmental disorders	4,470,675 (5.2)	15045 (61.5)	29.33 (23.51-36.6)	2.544
Injury, poisoning, & certain other consequences of external causes	7,559,439 (8.7)	1160 (4.7)	0.52 (0.43-0.63)	0.142
Diseases of the circulatory system	14,452,895 (16.7)	1965 (8.0)	0.44 (0.36-0.52)	0.233
Certain infections & parasitic diseases	7,128,154 (8.2)	445 (1.8)	0.21 (0.16-0.27)	0.234
Diseases of the respiratory system	6,918,933 (8.0)	970 (4.0)	0.47 (0.39-0.58)	0.149
Diseases of the skin & subcutaneous tissue	1,605,849 (1.9)	305 (1.2)	0.67 (0.5-0.89)	0.045
Endocrine, nutritional & metabolic diseases	3,761,624 (4.4)	1365 (5.6)	1.3 (1.07-1.58)	0.060
Diseases of the digestive system	8,581,113 (9.9)	685 (2.8)	0.26 (0.21-0.33)	0.238
Diseases of the musculoskeletal system & connective tissue	6,240,469 (7.2)	260 (1.1)	0.14 (0.1-0.19)	0.238
Diseases of the genitourinary system	4,085,434 (4.7)	290 (1.2)	0.24 (0.18-0.32)	0.167
Symptoms, signs, & abnormal clinical laboratory findings, not elsewhere classified	2,401,400 (2.8)	320 (1.3)	0.46 (0.35-0.62)	0.089
Diseases of the nervous system	2,226,485 (2.6)	360 (1.5)	0.57 (0.43-0.74)	0.070
Pregnancy, childbirth, & puerperium	11,157,270 (12.9)	685 (2.8)	0.19 (0.16-0.24)	0.302
Neoplasms	3,559,619 (4.1)	250 (1.0)	0.24 (0.18-0.33)	0.156
Diseases of the blood & blood-forming organs & certain disorders involving the immune mechanism	1,074,870 (1.2)	185 (0.8)	0.61 (0.43-0.86)	0.044
Factors influencing health status & contact with health services	908,655 (1.1)	130 (0.5)	0.5 (0.33-0.76)	0.051
Diseases of the eye & adnexa	65,820 (0.1)	5 (0.0)	0.27 (0.04-1.91)	0.020
Certain conditions originating in the perinatal period[c]	905 (0.0)	0.0 (0.0)	NA	NA
Congenital malformations, deformations & chromosomal abnormalities	109,530 (0.1)	5 (0.0)	0.16 (0.02-1.15)	0.030
Diseases of the ear & mastoid process	93,980 (0.1)	15 (0.1)	0.56 (0.18-1.77)	0.014
External causes of morbidity[c]	395 (0.0)	0.0 (0.0)	NA	NA
**Total number of hospital admissions**	**86,403,514** (98.9)	**24,445** (2.6)	NA	NA

Data source: Nationwide Inpatient Sample (NIS), 2017-2019

Abbreviations: NA, not applicable.

[a] = *Z59.0= homelessness; Z59.1= inadequate housing; Z59.2= discord with neighbors, lodgers, landlords; Z59.3= problems related to living in residential institution; Z59.8= other.*

[b] = *"Standardized difference" displays the absolute value of the difference in proportions divided by the standard error and is an indicator of effect size (Cohen d) (0.20-0.49 indicates small; 0.50 to 0.79 medium; and ≥0.80, large effect sizes)."*

[c] = *No standardized difference is reported for a hospitalization discharge diagnosis that had none of the particular housing instability Z59-code.*

© 2022 Rollings KA et al. *JAMA Network Open.*

eTable 7. Total Inpatient Days and Mean Length of Stay by Diagnosis Among Patients With and Without Housing Instability Codes Z59.0 (Homelessness), Z59.1 (Inadequate Housing), and Z59.2 (Discord With Neighbors, Lodgers, and Landlords)

Discharge Diagnosis	**Without Coded Housing Instability** days (%) (N=86,403,514)	**With Coded Housing Instability Z59-code** [a]					
		**Z59.0** days (%) (N=915,305)	**Stand. Diff.**[b]	**Z59.1** days (%) (N=5,030)	**Stand. Diff.**[b]	**Z59.2** days (%) (N=1,220)	**Stand. Diff.**[a]
Mental, behavioral and neurodevelopmental disorders	31,111,715 (7.6)	3544616 (59.0)	1.917	20440 (53.2)	1.722	9340 (93.0)	3.226
Injury, poisoning, and certain other consequences of external causes	42,977,862 (10.5)	398390 (6.6)	0.126	2285 (5.9)	0.148	130 (1.3)	0.300
Certain infections and parasitic diseases	48,729,037 (11.9)	376930 (6.3)	0.174	2360 (6.1)	0.177	30 (0.3)	0.358
Diseases of the circulatory system	71,429,857 (17.4)	338465 (5.6)	0.312	2375 (6.2)	0.296	75 (0.7)	0.440
Diseases of the respiratory system	34,258,750 (8.4)	225810 (3.8)	0.167	1540 (4.0)	0.157	55 (0.5)	0.282
Diseases of the skin and subcutaneous tissue	7,202,057 (1.8)	214790 (3.6)	0.138	1235 (3.2)	0.111	-	0.134
Endocrine, nutritional and metabolic diseases	15,839,212 (3.9)	169425 (2.8)	0.054	1055 (2.7)	0.058	40 (0.4)	0.180
Diseases of the digestive system	38,656,694 (9.4)	166540 (2.8)	0.229	645 (1.7)	0.265	10 (0.1)	0.319
Diseases of the musculoskeletal system and connective tissue	19,203,639 (4.7)	131340 (2.2)	0.119	470 (1.2)	0.164	50 (0.5)	0.198
Diseases of the nervous system	11,976,440 (2.9)	110970 (1.8)	0.064	2150 (5.6)	0.159	225 (2.2)	0.040
Diseases of the genitourinary system	16,894,038 (4.1)	82380 (1.4)	0.139	915 (2.4)	0.088	35 (0.3)	0.190
Pregnancy, childbirth, and puerperium	29,776,798 (7.3)	69975 (1.2)	0.236	1485 (3.9)	0.131	30 (0.3)	0.268
Neoplasms[c]	21,759,871 (5.3)	65775 (1.1)	0.189	415 (1.1)	0.189	0 (0.0)	NA
Symptoms, signs, and abnormal clinical laboratory findings, not elsewhere classified	8,401,415 (2.0)	63455 (1.1)	0.070	680 (1.8)	0.020	20 (0.2)	0.131
Diseases of the blood and blood-forming organs and certain disorders involving the immune mechanism[c]	4,727,569 (1.2)	20365 (0.3)	0.077	150 (0.4)	0.071	0 (0.0)	NA
Factors influencing health status and contact with health services	6,013,903 (1.5)	17910 (0.3)	0.098	225 (0.6)	0.073	0 (0.0)	0.122
Diseases of the eye and adnexa[c]	227,895 (0.1)	6565 (0.1)	0.023	0 (0.0)	NA	0 (0.0)	NA
Congenital malformations, deformations and chromosomal abnormalities[c]	549,135 (0.1)	1895 (0.0)	0.028	0 (0.0)	NA	0 (0.0)	NA
Diseases of the ear and mastoid process[c]	260,770 (0.1)	1475 (0.0)	0.016	0 (0.0)	NA	0 (0.0)	NA
External causes of morbidity[c]	1,505 (0.0)	20 (0.0)	<0.001	0 (0.0)	NA	0 (0.0)	NA
Certain conditions originating in the perinatal period[c]	3,695 (0.0)	0 (0.0)	NA	0 (0.0)	NA	0 (0.0)	NA
**Total inpatient days** (%)	**410,001,857** (98.5)	**6,007,089** (96.9)	NA	**38,425** (0.6)	NA	**10,040** (0.2)	NA
**Mean length of stay** (days)	**4.8** (4.7-4.8)	**6.6** (6.5 to 6.7)	0.285	**7.6** (6.6 to 8.7)	0.458	**8.2** (6.8 to 9.7)	0.552

Data source: Nationwide Inpatient Sample (NIS), 2017-2019

Abbreviations: NA, not applicable.

[a] = *Z59.0= homelessness; Z59.1= inadequate housing; Z59.2= discord with neighbors, lodgers, landlords; Z59.3= problems related to living in residential institution; Z59.8= other.*

[b] = *"Standardized difference" displays the absolute value of the difference in proportions divided by the standard error and is an indicator of effect size (Cohen d) (0.20-0.49 indicates small; 0.50 to 0.79 medium; and ≥0.80, large effect sizes)."*

[c] = *No standardized difference is reported for a hospitalization discharge diagnosis that had none of the particular housing instability Z59-code.*

© 2022 Rollings KA et al. *JAMA Network Open.*

eTable 8. Total Inpatient Days and Mean Length of Stay by Diagnosis Among Patients With and Without Housing Instability Codes Z59.3 (Problems Related to Living in a Residential Institution) and Z59.8 (Other Housing and Economic Problems)

**Discharge Diagnosis**	**Without Coded Housing Instability** days (%) (N=86,403,514)	**With Coded Housing Instability Z59-code**[a]			
		**Z59.3** days (%) (N=3,335)	**Standardized Difference**[b]	**Z59.8** days (%) (N=24,445)	**Standardized Difference**[b]
Mental, behavioral and neurodevelopmental disorders	31,111,715 (7.6)	12005 (48.2)	1.533	106120 (70.5)	2.375
Injury, poisoning, and certain other consequences of external causes	42,977,862 (10.5)	1400 (5.6)	0.159	6515 (4.3)	0.201
Certain infections and parasitic diseases	48,729,037 (11.9)	2400 (9.6)	0.070	2515 (1.7)	0.316
Diseases of the circulatory system	71,429,857 (17.4)	1405 (5.6)	0.311	8440 (5.6)	0.312
Diseases of the respiratory system	34,258,750 (8.4)	2440 (9.8)	0.052	4455 (3.0)	0.195
Diseases of the skin and subcutaneous tissue	7,202,057 (1.8)	275 (1.1)	0.050	1155 (0.8)	0.075
Endocrine, nutritional and metabolic diseases	15,839,212 (3.9)	455 (1.8)	0.106	4785 (3.2)	0.035
Diseases of the digestive system	38,656,694 (9.4)	1135 (4.6)	0.167	2915 (1.9)	0.256
Diseases of the musculoskeletal system and connective tissue	19,203,639 (4.7)	125 (0.5)	0.198	1440 (1.0)	0.176
Diseases of the nervous system	11,976,440 (2.9)	1390 (5.6)	0.158	3605 (2.4)	0.031
Diseases of the genitourinary system	16,894,038 (4.1)	1110 (4.5)	0.017	1225 (0.8)	0.166
Pregnancy, childbirth, and puerperium	29,776,798 (7.3)	25 (0.1)	0.276	2615 (1.7)	0.213
Neoplasms	21,759,871 (5.3)	165 (0.7)	0.207	1440 (1.0)	0.194
Symptoms, signs, and abnormal clinical laboratory findings, not elsewhere classified	8,401,415 (2.0)	280 (1.1)	0.065	1465 (1)	0.076
Diseases of the blood and blood-forming organs and certain disorders involving the immune mechanism	4,727,569 (1.2)	145 (0.6)	0.053	965 (0.6)	0.048
Factors influencing health status and contact with health services	6,013,903 (1.5)	85 (0.3)	0.094	780 (0.5)	0.079
Diseases of the eye and adnexa	227,895 (0.1)	60 (0.2)	0.079	20 (0.0)	0.018
Congenital malformations, deformations and chromosomal abnormalities[c]	549,135 (0.1)	0 (0.0)	NA	30 (0.0)	0.031
Diseases of the ear and mastoid process	260,770 (0.1)	15 (0.1)	0.001	30 (0.0)	0.017
External causes of morbidity[c]	1,505 (0.0)	0 (0.0)	NA	0 (0.0)	NA
Certain conditions originating in the perinatal period[c]	3,695 (0.0)	0 (0.0)	NA	0 (0.0)	NA
**Total inpatient days** (%)	**410,001,857** (98.5)	**24,915** (0.4)	NA	**150,515** (2.4)	NA
**Mean length of stay** (days)	**4.8** (4.7-4.8)	**7.5** (6.2 to 8.8)	0.432	**6.2** (5.7 to 6.6)	0.224

Data source: Nationwide Inpatient Sample (NIS), 2017-2019

Abbreviations: NA, not applicable.

[a] = *Z59.0= homelessness; Z59.1= inadequate housing; Z59.2= discord with neighbors, lodgers, landlords; Z59.3= problems related to living in residential institution; Z59.8= other.*

[b] = *"Standardized difference" displays the absolute value of the difference in proportions divided by the standard error and is an indicator of effect size (Cohen d) (0.20-0.49 indicates small; 0.50 to 0.79 medium; and ≥0.80, large effect sizes)."*

[c] = *No standardized difference is reported for a hospitalization discharge diagnosis that had none of the particular housing instability Z59-code.*

© 2022 Rollings KA et al. *JAMA Network Open.*

eTable 9. Cost (Millions, USD) of Hospitalization by Diagnosis Among Patients With and Without Housing Instability Codes Z59.0 (Homelessness), Z59.1 (Inadequate Housing), and Z59.2 (Discord With Neighbors, Lodgers, and Landlords)

Discharge Diagnosis	**Without Coded Housing Instability** Millions, USD (%) (N=86,403,514)	**With Coded Housing Instability Z59-code**[a]					
		**Z59.0** Millions, USD (%) (N=915,305)	**Stand. Diff.**[b]	**Z59.1** Millions, USD (%) (N=5,030)	**Stand. Diff.**[b]	**Z59.2** Millions, USD (%) (N=1,220)	**Stand. Diff.**[b]
Mental, behavioral and neurodevelopmental disorders	31,744.8 (2.7)	3412.0 (37.8)	2.069	22.2 (39.0)	2.232	8.9 (87.6)	5.214
Injury, poisoning, and certain other consequences of external causes	138,203.5 (11.9)	1069.9 (11.8)	<0.001	4.6 (8.0)	0.118	0.3 (3.1)	0.272
Diseases of the circulatory system	254,677.8 (21.8)	848.3 (9.4)	0.302	5.0 (8.8)	0.316	0.1 (0.6)	0.514
Certain infections and parasitic diseases	127,040.0 (10.9)	846.1 (9.4)	0.049	3.8 (6.7)	0.134	0.2 (1.6)	0.298
Diseases of the respiratory system	81,758.7 (7.0)	504.2 (5.6)	0.056	3.6 (6.3)	0.028	0.1 (0.5)	0.253
Diseases of the skin and subcutaneous tissue[c]	13,765.3 (1.2)	428.0 (4.7)	0.324	2.0 (3.6)	0.223	0.0 (0.0)	NA
Diseases of the digestive system	105,462.0 (9.0)	406.0 (4.5)	0.159	1.2 (2.1)	0.244	0.1 (0.8)	0.288
Endocrine, nutritional and metabolic diseases	42,263.5 (3.6)	346.1 (3.8)	0.011	2.3 (4.0)	0.019	0.1 (1.1)	0.136
Diseases of the musculoskeletal system and connective tissue	114,185.9 (9.8)	287.8 (3.2)	0.223	1.2 (2.2)	0.256	0.1 (1.2)	0.291
Diseases of the nervous system	28,243.7 (2.4)	211.1 (2.3)	0.006	2.5 (4.4)	0.132	0.2 (2.3)	0.006
Neoplasms[c]	79,056.1 (6.8)	158.1 (1.7)	0.201	1.1 (1.9)	0.195	0.0 (0.0)	NA
Diseases of the genitourinary system	38,380.6 (3.3)	156.4 (1.7)	0.088	1.6 (2.8)	0.027	0.1 (0.5)	0.157
Symptoms, signs, and abnormal clinical laboratory findings, not elsewhere classified	20,181.6 (1.7)	140.8 (1.6)	0.013	1.1 (2.0)	0.020	0.0 (0.5)	0.098
Pregnancy, childbirth, and puerperium	60,517.8 (5.2)	122.2 (1.4)	0.174	4.0 (7.1)	0.085	0.0 (0.3)	0.219
Diseases of the blood and blood-forming organs and certain disorders involving the immune mechanism[c]	11,965.6 (1.0)	46.5 (0.5)	0.051	0.3 (0.5)	0.047	0.0 (0.0)	NA
Factors influencing health status and contact with health services[c]	14,092.6 (1.2)	28.6 (0.3)	0.082	0.3 (0.6)	0.057	0.0 (0.0)	NA
Congenital malformations, deformations and chromosomal abnormalities[c]	3,345.5 (0.3)	6.1 (0.1)	0.041	0.0 (0.0)	NA	0.0 (0.0)	NA
Diseases of the eye and adnexa[c]	623.0 (0.1)	13.7 (0.2)	0.042	0.0 (0.0)	NA	0.0 (0.0)	NA
Certain conditions originating in the perinatal period[c]	10.9 (0.0)	0.0 (0.0)	NA	0.0 (0.0)	NA	0.0 (0.0)	NA
Diseases of the ear and mastoid process[c]	697.9 (0.1)	4.0 (0.0)	0.006	0.0 (0.0)	NA	0.0 (0.0)	NA
External causes of morbidity[c]	4.7 (0.0)	0.0 (0.0)	<0.001	0.0 (0.0)	NA	0.0 (0.0)	NA
**Total cost**	**1,166,221.5** (99.2)	**9,036.1** (97.0)	NA	**56.9** (0.6)	NA	**10.2** (0.1)	NA

Data source: Nationwide Inpatient Sample (NIS), 2017-2019

Abbreviations: NA, not applicable.

[a] = *Z59.0= homelessness; Z59.1= inadequate housing; Z59.2= discord with neighbors, lodgers, landlords; Z59.3= problems related to living in residential institution; Z59.8= other.*

[b] = *"Standardized difference" displays the absolute value of the difference in proportions divided by the standard error and is an indicator of effect size (Cohen d) (0.20-0.49 indicates small; 0.50 to 0.79 medium; and ≥0.80, large effect sizes)."*

[c] = *No standardized difference is reported for a hospitalization discharge diagnosis that had none of the particular housing instability Z59-code.*

© 2022 Rollings KA et al. *JAMA Network Open.*

**eTable 10. Cost (Millions, USD) of Hospitalization by Diagnosis Among Patients With and Without Housing Instability Codes Z59.3 (Problems Related to Living in a Residential Institution) and Z59.8 (Other Housing and Economic Problems)**					
**Discharge Diagnosis**	**Without Coded Housing Instability** Millions, USD (%) (N=86,403,514)	**With Coded Housing Instability Z59-code**[a]			
		**Z59.3** (%) (N=3,335)	**Standardized Difference**[b]	**Z59.8** (%) (N=24,445)	**Standardized Difference**[b]
Mental, behavioral and neurodevelopmental disorders	31,744.8 (2.7)	15.1 (35.3)	1.999	100.4 (49.8)	2.888
Injury, poisoning, and certain other consequences of external causes	138,203.5 (11.9)	3.4 (8.0)	0.120	13.9 (6.9)	0.154
Diseases of the circulatory system	254,677.8 (21.8)	3.6 (8.5)	0.324	22.2 (11)	0.262
Certain infections and parasitic diseases	127,040.0 (10.9)	5.5 (12.7)	0.060	5.6 (2.8)	0.260
Diseases of the respiratory system	81,758.7 (7.0)	5.7 (13.4)	0.249	10.0 (5.0)	0.080
Diseases of the skin and subcutaneous tissue	13,765.3 (1.2)	0.4 (0.9)	0.022	2.1 (1.0)	0.014
Diseases of the digestive system	105,462.0 (9.0)	2.2 (5.2)	0.135	7.4 (3.7)	0.187
Endocrine, nutritional and metabolic diseases	42,263.5 (3.6)	1.1 (2.6)	0.054	10.9 (5.4)	0.095
Diseases of the musculoskeletal system and connective tissue	114,185.9 (9.8)	0.2 (0.5)	0.313	4.8 (2.4)	0.249
Diseases of the nervous system	28,243.7 (2.4)	1.7 (4.0)	0.100	5.6 (2.8)	0.023
Neoplasms	79,056.1 (6.8)	0.5 (1.1)	0.225	4.1 (2.1)	0.188
Diseases of the genitourinary system	38,380.6 (3.3)	1.9 (4.5)	0.068	2.5 (1.2)	0.115
Symptoms, signs, and abnormal clinical laboratory findings, not elsewhere classified	20,181.6 (1.7)	0.7 (1.5)	0.015	3.0 (1.5)	0.020
Pregnancy, childbirth, and puerperium	60,517.8 (5.2)	0.0 (0.1)	0.229	4.9 (2.4)	0.125
Diseases of the blood and blood-forming organs and certain disorders involving the immune mechanism	11,965.6 (1.0)	0.4 (1.0)	0.007	2.5 (1.2)	0.019
Factors influencing health status and contact with health services	14,092.6 (1.2)	0.2 (0.5)	0.069	1.7 (0.9)	0.033
Congenital malformations, deformations and chromosomal abnormalities[c]	3,345.5 (0.3)	0 (0.0)	NA	0.1 (0.0)	0.049
Diseases of the eye and adnexa[c]	623.0 (0.1)	0.1 (0.3)	0.090	0.1 (0.0)	0.009
Certain conditions originating in the perinatal period[c]	10.9 (0.0)	0 (0.0)	NA	0 (0.0)	NA
Diseases of the ear and mastoid process[c]	697.9 (0.1)	0.0 (0.1)	0.023	0.1 (0.0)	0.013
External causes of morbidity[c]	4.7 (0.0)	0 (0.0)	NA	0 (0.0)	NA
**Total cost**	**1,166,221.5** (99.2)	**42.8** (0.5)	NA	**201.8** (2.2)	NA

Data source: Nationwide Inpatient Sample (NIS), 2017-2019

Abbreviations: NA, not applicable.

[a] = *Z59.0= homelessness; Z59.1= inadequate housing; Z59.2= discord with neighbors, lodgers, landlords; Z59.3= problems related to living in residential institution; Z59.8= other.*

[b] = *"Standardized difference" displays the absolute value of the difference in proportions divided by the standard error and is an indicator of effect size (Cohen d) (0.20-0.49 indicates small; 0.50 to 0.79 medium; and ≥0.80, large effect sizes)."*

[c] = *No standardized difference is reported for a hospitalization discharge diagnosis that had none of the particular housing instability Z59-code.*

© 2022 Rollings KA et al. *JAMA Network Open.*

eFigure. Most Common Reasons for Hospitalization Among Patients With and Without Coded Housing Instability

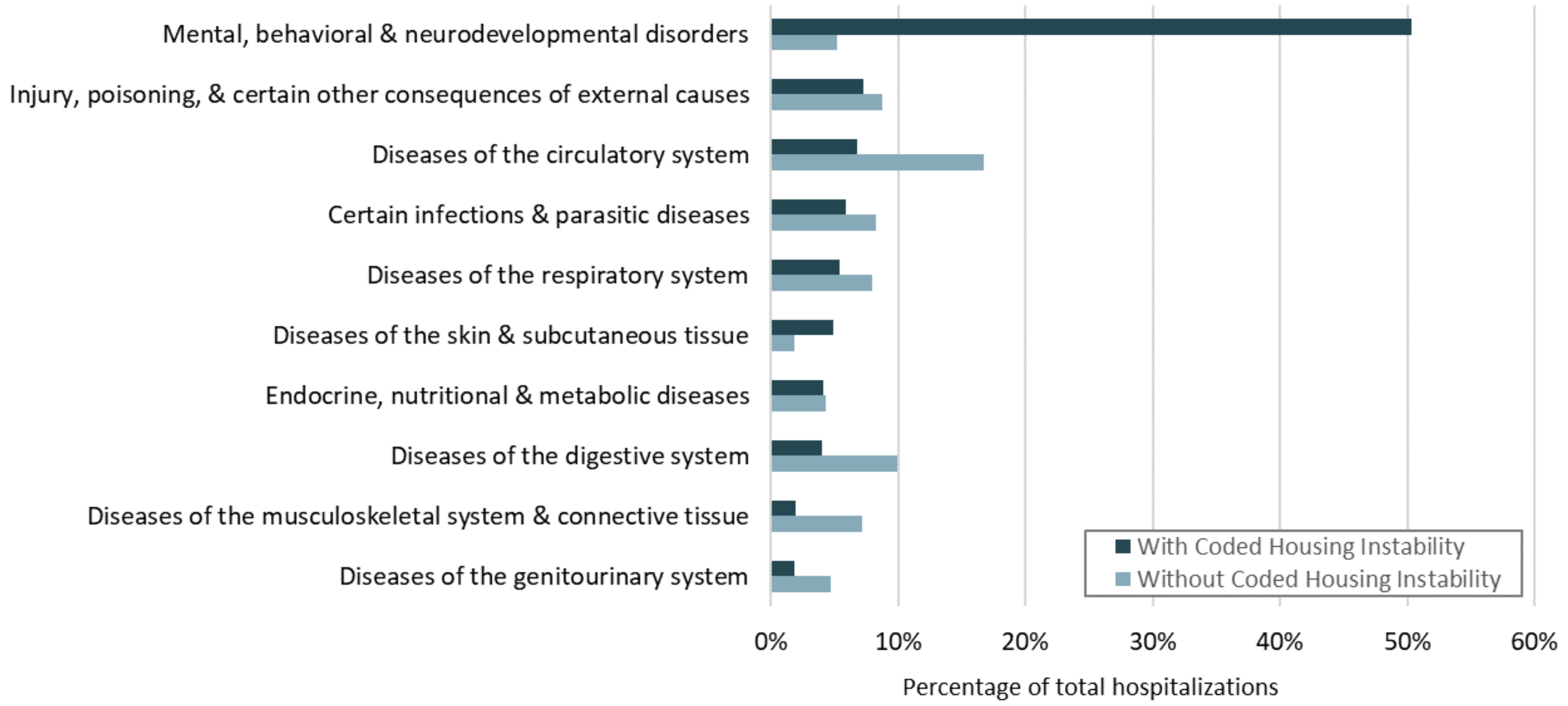

Data source: Nationwide Inpatient Sample (NIS), 2017-2019

The 10 most common primary diagnoses were identified for patients with and without coded housing instability using the International Classification of Diseases, 10th Revision, Clinical Modification (ICD-10-CM) codes for hospitalizations.

© 2022 Rollings KA et al. *JAMA Network Open.*
